# Tin (IV) Chloride-Promoted One-Pot Synthesis of Novel Tacrine Analogues

**DOI:** 10.3390/molecules16021878

**Published:** 2011-02-22

**Authors:** Huanan Hu, Liangfu Song, Qianqian Fang, Junjun Zheng, Zhiwei Meng, Yiting Luo

**Affiliations:** College of Pharmaceutical and Chemical Engineering, Taizhou University, Linhai 317000, China

**Keywords:** Alzheimer’s disease, tacrine, tin(IV) chloride, cyclocondensation

## Abstract

A facile synthesis of potential acetylcholinesterase (AChE) inhibitors, the tacrine analogues **3a-p**, has been accomplished by direct cyclocondensation of 1-aryl-4-cyano-5-aminopyrazole with β-ketoesters using tin(IV) chloride as catalyst. The structures of all the compounds have been confirmed by IR, ^1^H- and ^13^C-NMR.

## 1. Introduction 

Alzheimer’s disease (AD), the most common form of dementia among the elderly, is a progressive, degenerative disorder of the brain with a loss of memory and cognition [1]. Tacrine is a reversible inhibitor of acetylcholinesterase (AChE) that was launched in 1993 as the first drug for the treatment of AD [2]. The evaluation of the clinical effects of tacrine has shown efficacy in delaying the deterioration of the symptoms of AD, but the poor selectivity of this drug for AChE has resulted in a number of side effects, specially hepatotoxicity [3], and current research is focused on developing new AChE inhibitors with improved activity and reduced adverse side effects, therefore novel tacrine analogues have been reported [4,5,6,7,8,9,10,11,12,13,14,15,16,17,18]. 

Friedlander annulation is one of the simplest and most straightforward protocols for the preparation of quinoline derivatives [19,20]. Although it has been known for more than a century, it is still a hotspot of research. Herein, we report that the Friedlander annulation of 1-aryl-4-cyano-5-aminopyrazole with β-ketoesters using SnCl_4_ as catalyst afforded the novel tacrine analogues **3a-p** in good yields. 

## 2. Results and Discussion 

The main synthetic approach to tacrine analogues is the cyclocondensation of *o*-aminonitrile derivatives with ketones using anhydrous AlCl_3_ as catalyst, but that method gives low to moderate yields [4,5,6,7,8,9,10,11,12,13,14,15,16,17,18]. Cabrera and co-workers [21] have reported that the synthesis of 4-aminoquinolines from 2-aminobenzonitrile and β-dicarbonyl compounds could be accomplished using Lewis acids as catalysts. They found that SnCl_4_ was a very effective catalyst for the preparation of the target compounds compared with other Lewis acids.

For the investigation of reaction conditions for cyclocondensation of 1-aryl-4-cyano-5-amino-pyrazole with β-ketoesters, we chose the reaction of 1-phenyl-4-cyano-5-aminopyrazole (**1a**) with methyl acetoacetate as a model reaction. Because the choice of the catalyst played a crucial role, we initially studied the effect of several catalysts on the yields and found the use of anhydrous AlCl_3_, ZnCl_2_ and TiCl_4_ to be much less effective and tacrine analogue **3a** was obtained in less than 43% yield. Moreover, none of the product desired **3a** was obtained when we used CuCl and CuCl_2_ as catalysts. However, when a mixture of 1-phenyl-4-cyano-5-aminopyrazole (**1a**) and methyl acetoacetate in toluene was stirred under reflux in the presence of SnCl_4_, the reaction was complete within 3 h and after work up, the product **3a** was obtained in 78% yield (Table 1). 

A profound solvent effect on the reaction was observed. We initially studied the effect on the reaction of non-polar solvents, such as DCM, DCE, THF and toluene, in which SnCl_4_ is easily dissolved. Then we went on to study the polar solvent DMF. The results are summarized in Table 2. These results suggest that the solvent had a dramatic effect on the yields. Toluene was found to be the best solvent.

Under the optimized reaction conditions (SnCl_4_ as catalyst and anhydrous toluene as solvent, reflux for 3 h), a series of reactions between 1-aryl-4-cyano-5-aminopyrazoles and β-ketoesters were tested and a series of tacrine analogues **3** was thus prepared in good yields, regardless of the position of the group R^1^ on the aromatic ring. The results were summarized in Table 3.

**Scheme 2:**
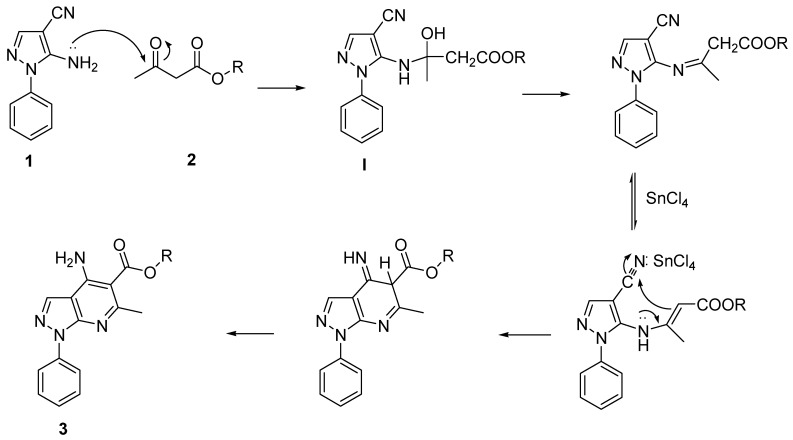
Proposed mechanism.

The mechanism of formation of tacrine analogue **3** can be explained by Scheme 2. The attack of the amino group of **1** onto the carbonyl carbon atom of **2** gave intermediate **I**, from which product **3** was obtained through the Friedlander reaction.

## 3. Experimental 

### 3.1. General

All melting points were determined on an XT-4A apparatus. TLC was performed using precoated silica gel GF_254_ (0.25 mm), column chromatography was performed using silica gel (200–300 mesh). The ^1^H- and ^13^C-NMR spectra were measured at 300 and 75 MHz, respectively, on a Bruker Advance 300 spectrometer at 25 °C, using TMS as internal standard. *J*-values are given in Hz. The IR spectra were taken on a Bruker Vector 55 spectrometer. 1-Aryl-4-cyano-5-aminopyrazoles **1** were prepared according to a reported procedure [22].

### 3.2. Typical procedure 

1-Aryl-4-cyano-5-aminopyrazole **1** (10 mmol) and SnCl_4_ (2.3 mL, 20 mmol) were added to a stirred solution of β-ketoester (10 mmol) in dry toluene (20 mL). The reaction mixture was stirred under nitrogen at room temperature for 30 min and then heated under reflux for 3 h. The reaction mixture was cooled and dispersed into water and titrated to pH 12–13 with a saturated aqueous solution of Na_2_CO_3_. After filtration, the filtrate was extracted three times with ethyl acetate, the organic layers were dried and evaporated at reduced pressure to give the solid product. The product was purified by silica gel column chromatography to give **3a-p**.

*Methyl 4-amino-6-methyl-1-phenyl-1H-pyrazolo[3,4-b]pyridine-5-carboxylate* (**3a**). Colorless solid. m.p. 125–126 °C. ^1^H-NMR (CDCl_3_) *δ*: 8.24 (d, *J* = 6.9 Hz, 2H, Ar-H), 8.04 (s, 1H, pyrazole H), 7.41–7.48 (m, 2H, Ar-H), 7.23–7.28 (m, 1H, Ar-H), 6.74 (br, 2H, -NH_2_), 3.90 (s, 3H, -OCH_3_), 2.80 (s, 3H, -CH_3_). ^13^C-NMR (CDCl_3_) *δ*: 169.9, 163.1, 151.8, 150.0, 138.7, 131.6, 128.8, 126.3, 121.6, 105.3, 101.2, 51.6, 28.1. MS-ESI (*m/z*, relative intensity, %): 285 (M^+^+3) (3), 284 (M^+^+2) (18), 283 (M^+^+1) (100). IR (KBr, cm^−1^) ν: 3,451, 3,330 (NH_2_), 3,111 (ArH), 2,976, 2,926 (CH_3_), 1,665 (C=O), 1,590, 1,495, 1,460 (C=N and aromatic ring skeleton vibration), 1,260 (O-CH_3_). 

*Methyl 4-amino-6-methyl-1-p-tolyl-1H-pyrazolo[3,4-b]pyridine-5-carboxylate* (**3b**). Colorless solid. m.p. 167–168 °C. ^1^H-NMR (CDCl_3_) *δ*: 8.08 (d, *J* = 7.2 Hz, 2H, Ar-H), 8.05 (s, 1H, pyrazole H), 7.25–7.29 (m, 2H, Ar-H), 6.74 (br, 2H, -NH_2_), 3.91 (s, 3H, -OCH_3_), 2.80 (s, 3H, pyridine-CH_3_), 2.38 (s, 3H, benzene-CH_3_). ^13^C-NMR (CDCl_3_) *δ*: 169.2, 162.1, 151.9, 149.5, 136.3, 135.4, 130.6, 128.9, 121.0, 104.7, 100.5, 51.5, 27.9, 20.8. MS-ESI (*m/z*, relative intensity, %): 299 (M^+^+3) (3), 298 (M^+^+2) (20), 297 (M^+^+1) (100). IR (KBr, cm^−1^) ν: 3,452, 3,333 (NH_2_), 3,110 (ArH), 2,976, 2,925 (CH_3_), 1,660 (C=O), 1,590, 1,495, 1,460 (C=N and aromatic ring skeleton vibration), 1,246 (O-CH_3_). 

*Methyl 4-amino-6-methyl-1-m-tolyl-1H-pyrazolo[3,4-b]pyridine-5-carboxylate* (**3c**). Colorless solid. m.p. 151–153 °C. ^1^H-NMR (CDCl_3_) *δ*: 8.07 (d, *J* = 7.6 Hz, 2H, Ar-H), 8.04 (s, 1H, pyrazole H), 7.33–7.39 (m, 1H, Ar-H), 7.11–7.16 (m, 1H, Ar-H), 6.72 (br, 2H, -NH_2_), 3.91 (s, 3H, -OCH_3_), 2.82 (s, 3H, pyridine-CH_3_), 2.40 (s, 3H, benzene-CH_3_). ^13^C-NMR (CDCl_3_) *δ*: 169.0, 162.1, 151.0, 149.5, 138.6, 131.4, 128.1, 126.2, 125.1, 121.5, 118.7, 104.9, 101.0, 51.8, 28.1, 21.0. MS-ESI (*m/z*, relative intensity, %): 299 (M^+^+3) (2), 298 (M^+^+2) (22), 297 (M^+^+1) (100). IR (KBr, cm^−1^) ν: 3,453, 3,332 (NH_2_), 3,112 (ArH), 2,978, 2,923 (CH_3_), 1,672 (C=O), 1,596, 1,497, 1,465 (C=N and aromatic ring skeleton vibration), 1,260 (O-CH_3_). 

*Methyl 4-amino-1-(4-chlorophenyl)-6-methyl-1H-pyrazolo[3,4-b]pyridine-5-carboxylate* (**3d**). Colorless solid. m.p. 160–162 °C. ^1^H-NMR (CDCl_3_) *δ*: 8.26 (d, *J* = 7.2 Hz, 2H, Ar-H), 8.04 (s, 1H, pyrazole H), 7.40–7.48 (m, 2H, Ar-H), 6.72 (br, 2H, -NH_2_), 3.91 (s, 3H, -OCH_3_), 2.81 (s, 3H, -CH_3_). ^13^C-NMR (CDCl_3_) *δ*: 168.8, 161.7, 151.2, 135.0, 131.7, 130.6, 127.9, 121.0, 118.2, 104.9, 100.4, 51.7, 28.0. MS-ESI (*m/z*, relative intensity, %): 320 (9), 319 (32), 317 (M^+^+1) (100). IR (KBr, cm^−1^) ν: 3,451, 3,330 (NH_2_), 3,111 (ArH), 2,976, 2,926 (CH_3_), 1,665 (C=O), 1,592, 1,494, 1,458 (C=N and aromatic ring skeleton vibration), 1,260 (O-CH_3_).

*Methyl 4-amino-1-(3-chlorophenyl)-6-methyl-1H-pyrazolo[3,4-b]pyridine-5-carboxylate* (**3e**). Colorless solid. m.p. 127–128 °C. ^1^H-NMR (CDCl_3_) *δ*: 8.36–8.40 (m, 1H, Ar-H), 8.27–8.31 (m, 1H, Ar-H), 8.05 (s, 1H, pyrazole H), 7.40–7.43 (m, 1H, Ar-H), 7.24–7.26 (m, 1H, Ar-H), 6.74 (br, 2H, -NH_2_), 3.90 (s, 3H), 2.82 (s, 3H, -CH_3_). ^13^C-NMR (CDCl_3_) *δ*: 168.9, 161.7, 151.1, 132.7, 131.5, 130.6, 130.1, 129.6, 128.4, 125.6, 121.7, 120.8, 118.4, 51.7, 28.0. MS-ESI (*m/z*, relative intensity, %): 319 (7), 318 (33), 317 (M^+^+1) (100). IR (KBr) ν: 3,453, 3,333 (NH_2_), 3,110 (ArH), 2,976, 2,924 (CH_3_), 1,668 (C=O), 1,592, 1,496, 1,461 (C=N and aromatic ring skeleton vibration), 1,258 (O-CH_3_).

*Methyl 4-amino-1-(2-chlorophenyl)-6-methyl-1H-pyrazolo[3,4-b]pyridine-5-carboxylate* (**3f**). Color-less solid. m.p. 154–155 °C. ^1^H-NMR (CDCl_3_) *δ*: 8.12 (s, 1H, Ar-H), 8.05 (s, 1H, pyrazole H), 7.55–7.58 (m, 1H, Ar-H), 7.39–7.42 (m, 2H, Ar-H), 6.74 (br, 2H, -NH_2_), 3.90 (s, 3H, -OCH_3_), 2.80 (s, 3H, -CH_3_). ^13^C-NMR (CDCl_3_) *δ*: 169.2, 162.8, 151.4, 151.3, 135.9, 131.7, 132.1, 130.5, 129.4, 129.1, 126.4, 103.1, 100.9, 51.6, 27.9. MS-ESI (*m/z*, relative intensity, %): 319 (8), 318 (33), 317 (M^+^+1) (100). IR (KBr, cm^−1^) ν: 3,451, 3,330 (NH_2_), 3,112 (ArH), 2,975, 2,923 (CH_3_), 1,665 (C=O), 1,591, 1,495, 1,458 (C=N and aromatic ring skeleton vibration), 1,262 (O-CH_3_).

*Methyl 4-amino-6-methyl-1-(4-nitrophenyl)-1H-pyrazolo[3,4-b]pyridine-5-carboxylate* (**3g**). Yellowish solid. m.p. 190–192 °C. ^1^H-NMR (DMSO) *δ*: 8.62–8.74 (m, 3H, Ar-H and pyrazole H), 8.37–8.40 (m, 2H, Ar-H), 7.84 (br, 2H, -NH_2_), 3.92 (s, 3H, -OCH_3_), 2.79 (s, 3H, -CH_3_). ^13^C-NMR (DMSO) *δ*: 168.0, 161.4, 151.3, 150.9, 144.6, 136.6, 125.1, 119.8, 118.5, 105.0, 102.4, 51.8, 29.0. MS-ESI (*m/z*, relative intensity, %): 330 (M^+^+3) (3), 329 (M^+^+2) (18), 328 (M^+^+1) (100). IR (KBr, cm^−1^) ν: 3,451, 3,330 (NH_2_), 3,111 (ArH), 2,976, 2,926 (CH_3_), 1,680 (C=O), 1,610, 1,543, 1,508 (C=N and aromatic ring skeleton vibration), 1,272 (O-CH_3_). 

*Methyl 4-amino-1-(2,4-dinitrophenyl)-6-methyl-1H-pyrazolo[3,4-b]pyridine-5-carboxylate* (**3h**). Yellowish solid. m.p. 202–204 °C. ^1^H-NMR (DMSO) *δ*: 8.86 (s, 1H, Ar-H), 8.69-8.73 (m, 1H, Ar-H), 8.52 (s, 1H, pyrazole H), 8.41–8.45 (m, 1H, Ar-H), 7.76 (br, 2H, -NH_2_), 3.91 (s, 3H, -OCH_3_), 2.80 (s, 3H, -CH_3_). ^13^C-NMR (DMSO) *δ*: 167.8, 162.2, 151.4, 150.7, 137.4, 134.9, 128.6, 128.4, 126.3, 126.1, 126.0, 121.7, 119.5, 51.8, 28.2. MS-ESI (*m/z*, relative intensity, %): 375 (M^+^+3) (5), 374 (M^+^+2) (21), 373 (M^+^+1) (100). IR (KBr, cm^−1^) ν: 3,453, 3,332 (NH_2_), 3,112 (ArH), 2,979, 2,933 (CH_3_), 1,679 (C=O), 1,612, 1,550, 1,515 (C=N and aromatic ring skeleton vibration), 1,277 (O-CH_3_).

*Ethyl 4-amino-6-methyl-1-phenyl-1H-pyrazolo[3,4-b]pyridine-5-carboxylate* (**3i**). Colorless solid. m.p. 130–131 °C. ^1^H-NMR (CDCl_3_) *δ*: 8.25 (d, *J* = 7.8 Hz, 2H, Ar-H), 8.04 (s, 1H, pyrazole H), 7.46–7.50 (m, 2H, Ar-H), 7.25–7.31 (m, 1H, Ar-H), 6.76 (br, 2H), 4.39 (q, *J* = 7.2 Hz, 2H, -CH_2_CH_3_), 2.81 (s, 3H, -CH_3_), 1.42 (t, *J* = 7.2 Hz, 3H, -CH_2_CH_3_). ^13^C-NMR (CDCl_3_) *δ*: 169.2, 162.5, 151.4, 150.2, 139.4, 131.7, 128.9, 126.1, 121.4, 105.1, 101.5, 60.7, 28.4, 14.3. MS-ESI (*m/z*, relative intensity, %): 299 (M^+^+3) (3), 298 (M^+^+2) (19), 297 (M^+^+1) (100). IR (KBr, cm^−1^) ν: 3,453, 3,333 (NH_2_), 3,110 (ArH), 2,976, 2,926 (CH_2_ and CH_3_), 1,665 (C=O), 1,592, 1,494, 1,460 (C=N and aromatic ring skeleton vibration), 1,260 (O-CH_3_). 

*Ethyl 4-amino-6-methyl-1-p-tolyl-1H-pyrazolo[3,4-b]pyridine-5-carboxylate* (**3j**). Colorless solid. m.p. 173–174 °C. ^1^H-NMR (CDCl_3_) *δ*: 8.09 (d, *J* = 6.6 Hz, 2H, Ar-H), 8.04 (s, 1H, pyrazole H), 7.25–7.29 (m, 2H, Ar-H), 6.72 (br, 2H, -NH_2_), 4.40 (q, *J* = 7.2 Hz, 2H, -CH_2_CH_3_), 2.81 (s, 3H, pyridine-CH_3_), 2.39 (s, 3H, benzene-CH_3_), 1.43 (t, *J* = 7.2 Hz, 3H, -CH_2_CH_3_). ^13^C-NMR (CDCl_3_) *δ*: 168.9, 162.0, 151.0, 149.7, 136.5, 135.5, 130.9, 129.1, 121.1, 104.5, 100.9, 60.3, 28.0, 20.6, 13.9. MS-ESI (*m/z*, relative intensity, %): 313 (M^+^+3) (2.5), 312 (M^+^+2) (22), 311 (M^+^+1) (100). IR (KBr, cm^−1^) ν: 3,453, 3,335 (NH_2_), 3,112 (ArH), 2,977, 2,930 (CH_2_ and CH_3_), 1,658 (C=O), 1,590, 1,492, 1,460 (C=N and aromatic ring skeleton vibration), 1,260 (O-CH_3_). 

*Ethyl 4-amino-6-methyl-1-m-tolyl-1H-pyrazolo[3,4-b]pyridine-5-carboxylate* (**3k**). Colorless solid. m.p. 148–149 °C. ^1^H-NMR (CDCl_3_) *δ*: 8.08 (d, *J* = 8.7 Hz, 2H, Ar-H), 8.01 (s, 1H, pyrazole H), 7.38 (t, *J* = 7.8 Hz, 1H, Ar-H), 7.11 (d, *J* = 7.5 Hz, 1H, Ar-H), 6.72 (br, 2H, -NH_2_), 4.40 (q, *J* = 7.2 Hz, 2H, -CH_2_CH_3_), 2.82 (s, 3H, pyridine-CH_3_), 2.45 (s, 3H, benzene-CH_3_), 1.43 (t, *J* = 7.2 Hz, 3H, -CH_2_CH_3_). ^13^C-NMR (CDCl_3_) *δ*: 168.8, 162.1, 150.9, 149.6, 138.5, 131.1, 128.3, 126.6, 125.6, 121.7, 118.3, 104.6, 101.1, 60.4, 28.0, 21.2, 13.9. MS-ESI (*m/z*, relative intensity, %): 313 (M^+^+3) (2), 312 (M^+^+2) (20), 311 (M^+^+1) (100). IR (KBr, cm^−1^) ν: 3,453, 3,336 (NH_2_), 3,111 (ArH), 2,976, 2,926 (CH_2_ and CH_3_), 1,662 (C=O), 1,590, 1,494, 1,462 (C=N and aromatic ring skeleton vibration), 1,262 (O-CH_3_). 

*Ethyl 4-amino-1-(4-chlorophenyl)-6-methyl-1H-pyrazolo[3,4-b]pyridine-5-carboxylate* (**3l**). Colorless solid. m.p. 143–144 °C. ^1^H-NMR (CDCl_3_) *δ*: 8.27 (d, *J* = 6.6 Hz, 2H, Ar-H), 8.04 (s, 1H, pyrazole H), 7.43–7.49 (m, 2H, Ar-H), 6.74 (br, 2H, -NH_2_), 4.40 (q, *J* = 7.2 Hz, 2H, -CH_2_CH_3_), 2.81 (s, 3H, -CH_3_), 1.43 (t, *J* = 7.2 Hz, 3H, -CH_2_CH_3_). ^13^C-NMR (CDCl_3_) *δ*: 168.9, 162.0, 151.0, 135.3, 131.6, 130.9, 128.1, 121.1, 118.5, 104.5, 100.9, 60.3, 28.0, 13.9. MS-ESI (*m/z*, relative intensity, %): 334 (8), 333 (33), 331 (M^+^+1) (100). IR (KBr, cm^−1^) ν: 3,453, 3,338 (NH_2_), 3,113 (ArH), 2,973, 2,925 (CH_2_ and CH_3_), 1,660 (C=O), 1,593, 1,457 (C=N and aromatic ring skeleton vibration), 1,260 (O-CH_3_). 

*Ethyl 4-amino-1-(3-chlorophenyl)-6-methyl-1H-pyrazolo[3,4-b]pyridine-5-carboxylate* (**3m**). Colorless solid. m.p. 105–106 °C. ^1^H-NMR (CDCl_3_) *δ*: 8.37–8.42 (m, 1H, Ar-H), 8.28–8.33 (m, 1H, Ar-H), 8.04 (s, 1H, pyrazole H), 7.41–7.46 (m, 1H, Ar-H), 7.23–7.27 (m, 1H, Ar-H), 6.73 (br, 2H, -NH_2_), 4.41 (q, *J* = 7.2 Hz, 2H, -CH_2_CH_3_), 2.83 (s, 3H, -CH_3_), 1.44 (t, *J* = 7.2 Hz, 3H, -CH_2_CH_3_). ^13^C-NMR (CDCl_3_) *δ*: 168.7, 161.9, 150.9, 132.9, 131.6, 130.6, 130.2, 129.5, 128.6, 125.4, 121.8, 120.6, 118.5, 60.4, 27.9, 13.8. MS-ESI (*m/z*, relative intensity, %): 334 (6), 333 (31), 331 (M^+^+1) (100). IR (KBr, cm^−1^) ν: 3,453, 3,337 (NH_2_), 3,111 (ArH), 2,974, 2,928 (CH_2_ and CH_3_), 1,661 (C=O), 1,592, 1,490, 1,458 (C=N and aromatic ring skeleton vibration), 1,258 (O-CH_3_). 

*Ethyl 4-amino-1-(2-chlorophenyl)-6-methyl-1H-pyrazolo[3,4-b]pyridine-5-carboxylate* (**3n**). Colorless solid. m.p. 164–165 °C. ^1^H-NMR (CDCl_3_) *δ*: 8.11 (s, 1H, Ar-H), 8.04 (s, 1H, pyrazole H), 7.51–7.57 (m, 1H, Ar-H), 7.38–7.42 (m, 2H, Ar-H), 6.83 (br, 2H, -NH_2_), 4.40 (q, *J* = 7.2 Hz, 2H, -CH_2_CH_3_), 2.72 (s, 3H), 1.41 (t, *J* = 7.2 Hz, 3H, -CH_2_CH_3_). ^13^C-NMR (CDCl_3_) *δ*: 168.8, 162.5, 151.1, 150.9, 135.4, 131.9, 131.8, 130.2, 129.6, 129.4, 126.9, 103.4, 101.2, 60.3, 27.7, 13.9. MS-ESI (*m/z*, relative intensity, %): 334 (5), 333 (32), 331 (M^+^+1) (100). IR (KBr, cm^−1^) ν: 3,453, 3,338 (NH_2_), 3,112 (ArH), 2,973, 2,930 (CH_2_ and CH_3_), 1,660 (C=O), 1,590, 1,493, 1,457 (C=N and aromatic ring skeleton vibration), 1,263 (O-CH_3_). 

*Ethyl 4-amino-6-methyl-1-(4-nitrophenyl)-1H-pyrazolo[3,4-b]pyridine-5-carboxylate* (**3o**). Yellowish solid. m.p. 197–199 °C. ^1^H-NMR (DMSO) *δ*: 8.62–8.74 (m, 3H, Ar-H and pyrazole H), 8.36–8.41 (m, 2H, Ar-H), 7.84 (br, 2H, -NH_2_), 4.32 (q, *J* = 7.2 Hz, 2H, -CH_2_CH_3_), 2.67 (s, 3H, -CH_3_), 1.33 (t, *J* = 7.2 Hz, 3H, -CH_2_CH_3_). ^13^C-NMR (DMSO) *δ*: 167.8, 161.2, 151.0, 150.5, 144.4, 136.3, 124.9, 119.6, 118.9, 105.4, 102.6, 60.6, 29.3, 14.2. MS-ESI (*m/z*, relative intensity, %): 344 (M^+^+3) (2.5), 343 (M^+^+2) (18), 342 (M^+^+1) (100). IR (KBr, cm^−1^) ν: 3,453, 3,333 (NH_2_), 3,112 (ArH), 2,978, 2,930 (CH_2_ and CH_3_), 1,678 (C=O), 1,616, 1,545, 1,512 (C=N and aromatic ring skeleton vibration), 1,275 (O-CH_3_). 

*Ethyl 4-amino-1-(2,4-dinitrophenyl)-6-methyl-1H-pyrazolo[3,4-b]pyridine-5-carboxylate* (**3p**). Yellowish solid. m.p. 205–206 °C. ^1^H-NMR (DMSO) *δ*: 8.86 (s, 1H, Ar-H), 8.69–8.73 (m, 1H, Ar-H), 8.52 (s, 1H, pyrazole H), 8.43 (d, *J* = 9.0 Hz, 1H, Ar-H), 7.74 (br, 2H, -NH_2_), 4.39 (q, *J* = 7.2 Hz, 2H, -CH_2_CH_3_), 2.66 (s, 3H, -CH_3_), 1.39 (t, *J* = 7.2 Hz, 3H, -CH_2_CH_3_). ^13^C-NMR (DMSO) *δ*: 167.8, 162.2, 151.4, 150.7, 137.4, 134.9, 128.6, 128.4, 126.3, 126.1, 126.0, 121.7, 119.5, 60.2, 27.9, 14.0. MS-ESI (m/z, relative intensity, %): 389 (M^+^+3) (4), 388 (M^+^+2) (19), 387 (M^+^+1) (100). IR (KBr, cm^−1^) ν: 3,453, 3,331 (NH_2_), 3,113 (ArH), 2,979, 2,932 (CH_2_ and CH_3_), 1,680 (C=O), 1,617, 1,545, 1,512 (C=N and aromatic ring skeleton vibration), 1,272 (O-CH_3_).

## 4. Conclusions

We have developed an efficient catalyst system using SnCl_4_ in anhydrous toluene for the reaction between *o*-aminonitriles containing pyrazole moieties with β-ketoesters. A series of novel tacrine analogues **3a-p** has been thus synthesized and their spectroscopic characterization and structural features have been presented.

## Figures and Tables

**Scheme 1:**
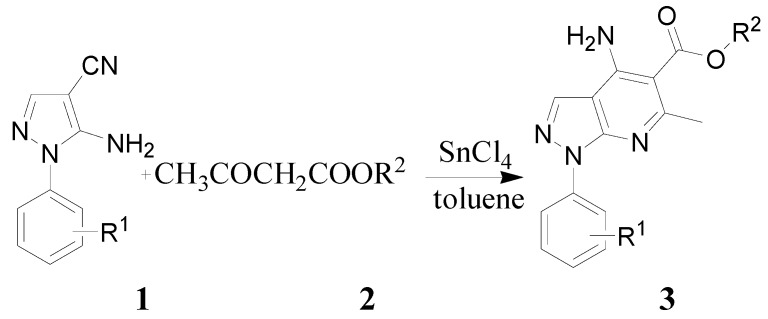
Cyclocondensation of 4-cyano-5-aminopyrazoles and β-ketoesters.

**Table 1:** Effect of the catalyst on the yields. *^a^*

Entry	Catalyst	Time(h)	Yield(%)*^b^*
1 2 3 4 5 6	CuCl CuCl_2_ AlCl_3_ ZnCl_2_ TiCl_4_ SnCl_4_	3 3 3 3 3 3	0 0 36 23 43 78

*^a^* All reactions were carried out using **1a** (10 mmol), methyl acetoacetate (10 mmol), catalyst (20 mmol) and toluene (20 mL), reflux for 3 h; *^b^* Isolated yields.

**Table 2:** Effect of the solvent on the yields. *^a^*

Entry	Solvent	Time (h)	Yield (%)*^b^*
1 2 3 4 5	DCM DCE THF Toluene DMF	3 3 3 3 3	53 68 51 78 0

*^a^* All reactions were carried out using **1a** (10 mmol), methyl acetoacetate (10 mmol), SnCl_4_ (20 mmol) and solvent (20 mL), reflux for 3 h; *^b^* Isolated yields.

**Table 3:** Cyclocondensation of substituted *o*-aminobenzonitrile and β-ketoesters. *^a^*

Entry	R^1^	R^2^	Product(3)	Yield(%)*^b^*
1 2 3 4 5 6 7 8 9 10 11 12 13 14 15 16	H 4-CH_3_ 3-CH_3_ 4-Cl 3-Cl 2-Cl 4-NO_2_ 2,4-NO_2_ H 4-CH_3_3 -CH_3_ 4-Cl 3-Cl 2-Cl 4-NO_2_ 2,4-NO_2_	Me Me Me Me Me Me Me Me Et Et Et Et Et Et Et Et	3a 3b 3c 3d 3e 3f 3g 3h 3i 3j 3k 3l 3m 3n 3o 3p	78 75 70 74 72 65 62 58 76 74 71 75 73 64 60 57

*^a^* All reactions were carried out using **1a** (10 mmol), β-ketoesters (10 mmol), SnCl_4_ (20 mmol) and toluene (20 mL), reflux for 3 h; *^b^* Isolated yields.
